# The Correlation Between the Ratio of ALT to qHBsAg and the Recompensation of HBV-Related Cirrhosis Patients: A Retrospective Cohort Study Based on the Baveno VII Criteria

**DOI:** 10.5152/tjg.2025.25039

**Published:** 2025-10-20

**Authors:** Yaping Xu, Yiheng Zhang, Shuning Jiao, Chunlei Lin, Qian Ye, Yan Wang

**Affiliations:** Department of Infectious Diseases and Hepatology, The Second Hospital of Shandong University, Jinan, China

**Keywords:** ALT/logqHBsAg, Baveno VII criteria, HBV, recompensation, cirrhosis

## Abstract

**Background/Aims:**

Patients with hepatitis B–related decompensated cirrhosis may achieve recompensation. The alanine aminotransferase (ALT) to quantitative hepatitis B surface antigen (qHBsAg) ratio is a novel predictor for hepatitis B surface antigen (HBsAg) seroclearance. This study evaluates its utility in predicting recompensation based on Baveno VII criteria.

**Materials and Methods:**

Decompensated hepatitis B–related cirrhosis patients were recruited and received antiviral treatment for at least 12 months. Classification of these participants into the decompensated and recompensated groups was established according to the Baveno VII criteria. Logistic regression and subgroup analysis assessed the correlation between the ratio of ALT to qHBsAg at baseline and cirrhotic recompensation.

**Results:**

A total of 136 patients were involved in this study; 80 (58.8%) patients achieved recompensation. Univariate analysis associated recompensation with sex, age, ALT, aspartate aminotransferase, hepatitis B virus DNA (HBV DNA), qHBsAg, and ALT/logqHBsAg. Multivariate analysis confirmed that higher ALT/logqHBsAg independently predicted greater recompensation likelihood (odds ratio [OR] = 1.01, 95% confidence interval [CI] = 1.00~1.02; *P *= .027). Categorically, ALT/logqHBsAg > 23.48 significantly increased recompensation probability (OR = 3.21, 95% CI = 1.31~7.90, *P *= .011). Subgroup analyses across 7 pre-specified subgroups (sex, age, hepatitis B e antigen, HBV DNA, Child–Pugh grade, Model for End-Stage Liver Disease score, qHBsAg) demonstrated consistent relationships. Enhanced predictive power was observed in Child–Pugh class C versus classes A/B and in males versus females.

**Conclusion:**

Elevated baseline ALT/logqHBsAg predicts a higher likelihood of hepatic recompensation in hepatitis B–related cirrhosis under Baveno VII criteria.

**Main Points**

- About 58.8% of patients with decompensated cirrhosis caused by the hepatitis B virus achieved recompensation following standardized antiviral treatment.
- When the alanine aminotransferase (ALT) ALT/logqHBsAg value is higher, there is a greater possibility of recompensation.
- This ratio exhibits better predictive performance in Child–Pugh class C and male patients.
- The ALT/logqHBsAg is not only useful for predicting hepatitis B surface antigen seroclearance but also for predicting the occurrence of recompensation.

## Introduction

Hepatitis B virus (HBV) is a hepadnavirus with unique replication characteristics, acting as the principal agent responsible for chronic hepatitis B (CHB), which may present with mild symptoms or cause severe liver damage.^1^ The global prevalence of CHB is about 316 million.^2^ Over time, about 15%-40% of untreated chronic hepatitis B patients develop cirrhosis or liver cancer, with a higher risk for men than for women.^3^ This has placed a huge economic burden on global health and sanitation systems.

Based on clinical characteristics, cirrhosis can be classified into the compensated stage (without clinical symptoms) and the decompensated stage (accompanied by ascites, variceal bleeding, and hepatic encephalopathy).^4^ However, it can be observed that some patients who have progressed to the decompensated stage of cirrhosis may experience an improvement and gradual stabilization of liver function after targeted etiological treatment, achieving disease stability or reversal to a state of “recompensation” and even a cirrhosis-free condition.^5^^-^^7^

Previous studies had shown that higher alanine aminotransferase (ALT) or aspartate aminotransferase (AST) levels at baseline were associated with hepatic recompensation.^8^^,^^9^ Recently, the ALT/qHBsAg (quantitative hepatitis B surface antigen) can be used to predict long-term hepatitis B surface antigen (HBsAg) seroclearance following the discontinuation of entecavir. The results demonstrated that the ratio has a good correlation and predictive ability for HBsAg seroclearance.^10^

However, there is no relevant research on the relationship between this ratio and hepatic recompensation, and it remains unclear. Considering the ALT/qHBsAg serves as a straightforward indicator of the equilibrium in the host’s immune response to HBsAg, in line with the Baveno VII criteria, this study delved into how the ratio was related to the recompensation of patients with HBV-related cirrhosis.

## Materials and Methods

### Study Population

This is a single-center retrospective cohort study of patients with HBV-related cirrhosis conducted between January 2016 and December 2023 at the Second Hospital of Shandong University. All the participants commenced first-line treatment using nucleos(t)ide analogues, either within 6 months before being enrolled or right after enrollment. The ethics committee at the Second Hospital of Shandong University gave its approval for this study. The ethics committee-approved account: NO: KYLL 2024 920. Approval date: November 1, 2024. Over the entire follow-up duration, the patients were sorted into 2 different groups: the decompensated group, which included patients who had been through at least 1 decompensation event and did not succeed in achieving recompensation during the study; and the recompensated group, comprising patients with decompensation who achieved recompensation in line with the Baveno VII criteria after treatment.^9^ In this study, the use of existing medical records or databases means that the data were not specifically collected for research purposes. Therefore, it was not possible to obtain informed consent at the beginning.

### Inclusion and Exclusion Criteria

The criteria for inclusion: (1) age ≥20 years old; (2) diagnosis of HBV-related decompensated cirrhosis made according to clinical, radiological, or histological findings. The criteria for exclusion: (1) co-existing other chronic liver diseases: autoimmune liver disease, alcoholic liver disease, hepatitis C, drug-induced liver disease, or severe non-alcoholic fatty liver disease; (2) occurrence of hepatocellular carcinoma (HCC) or death within 6 months after enrollment; (3) a previous history of HCC; (4) follow-up duration less than 48 weeks.

### Data Collection

For this study, all relevant data were sourced from the hospital’s electronic documentation of patients’ medical histories. Baseline data were represented by the data collected at the beginning of antiviral treatment, including age, gender, platelet count (PLT), neutrophil-to-lymphocyte ratio (NLR), ALT, AST, gamma-glutamyl transpeptidase (GGT), alkaline phosphatase (ALP), total bilirubin (TBIL), albumin (ALB), alpha-fetoprotein (AFP), international normalized ratio (INR), hepatitis B e antigen (HBeAg) status, qHBsAg, and serum HBV DNA (<20 IU/mL). Child–Pugh score, Fibrosis-4 Index (FIB-4), model for end-stage liver disease (MELD) score, and ALT/logqHBsAg were further calculated using relevant formulas. Moreover, we amassed data on the complications that are related to cirrhosis, including ascites, hepatic encephalopathy, and bleeding from esophageal varices, along with comorbid diseases such as diabetes.

### Definitions and Study Outcomes

Any one of the following factors led to the diagnosis of liver cirrhosis: clinical symptoms related to portal hypertension; imaging findings on ultrasonography, computed tomography, or magnetic resonance imaging indicative of cirrhosis; histopathological evidence. Recompensation was defined by satisfying each of the criteria: (1) sustained viral suppression for hepatitis B, characterized by undetectable serum HBV DNA (<20 IU/mL) or HBsAg seroclearance; (2) resolution of ascites (with discontinuation of diuretics) and hepatic encephalopathy (with discontinuation of lactulose/rifaximin), and no recurrent variceal bleeding for at least 12 months; and (3) stable improvement in liver function tests, indicated by a MELD score <10 and/or Child–Pugh Class A.^9^ The main endpoint was the incidence of recompensation. The data were censored at the earliest time point of the last follow-up, the diagnosis of HCC, or the patient’s death.

### Statistical Analysis

Categorical variables are presented in the form of the number (%), followed by a comparison using the chi-squared test. Continuous variables are displayed as the median (interquartile range) or the mean (±SD), and compared groups using either the Kruskal–Wallis test or the *t*-test. Two groups were formed by separating ALT/logqHBsAg based on the cut-off value of 23.48 (The ideal cut-off for the ALT/logqHBsAg ratio was determined by maximizing the Youden’s index). To explore the association between ALT/logqHBsAg and the recompensation of HBV-related cirrhosis patients, univariate and multivariate logistic analysis were used, including a minimally adjusted model (Model 1; adjusted for gender and age), partially adjusted model (Model 2, adjusted for gender, age, complete blood count, liver function, and tumor markers) and a fully adjusted model (Model 3, adjusted for gender, age, complete blood count, liver function, tumor markers, coagulation parameters, scores, and comorbidities). Effect sizes (OR) with 95% confidence intervals (CIs) were recorded. Subgroup analysis was also conducted according to the following 7 factors: sex, age, HBeAg status, HBV DNA, MELD score, Child–Pugh grade, and qHBsAg.

The Free Statistics analysis platform (Version 1.8, Beijing, China) (Company; city, Country) and R Statistical Software (Version 4.2.2, http://www.R-project.org, The R Foundation) (Company; city, Country) were used to conduct all the analyses. Statistical significance was defined as being indicated by a 2-tailed *P-*values < .05.

## Results

### Baseline Characteristics

**Figure 1.**
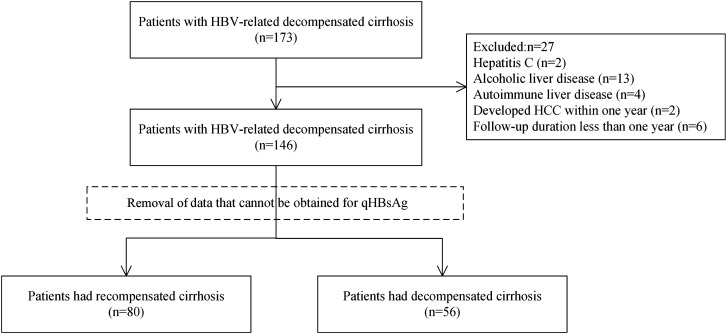
Flowchart of the study.

Initially, 173 patients who had HBV-related decompensated cirrhosis were screened. According to the pre-established exclusion criteria, 146 patients were screened out. Then, patients whose baseline qHBsAg data could not be obtained were further excluded. Ultimately, 136 of these patients were included in the cohort (Figure 1), among them, ALT/logqHBsAg > 23.48 accounted for 77 patients (56.62%). At baseline, the patients’ mean age was 54.6 years, and 61.8% of them were male. The mean ALT was 144.9 U/L, AST was 147.6 U/L, and HBV DNA was 5.3 log10 IU/mL. The decompensated events consisted of ascites (117, 86%), esophageal variceal bleeding (14, 10.3%), and hepatic encephalopathy (17, 12.5%). The baseline characteristics are presented in more detail in Table 1. In addition, baseline characteristics were compared between patients who achieved recompensation and those who did not (Supplementary Table 1).

### Factors Associated with Hepatic Recompensation

A total of 80 patients (58.82%) achieved hepatic recompensation by the end of the study. This study found that in the univariate analysis, when the levels of ALT, AST, INR, HBV DNA, qHBsAg, and ALT/logqHBsAg were higher, there was a stronger possibility that recompensation would occur; whereas old age and male sex were notably linked with a lessened likelihood of recompensation (Table 2).

Three multivariate analysis models were established: the minimally adjusted model (Model 1, adjusting for gender and age), the partially adjusted model (Model 2, adjusting for gender, age, complete blood count, liver function, and tumor markers), and the fully adjusted model (Model 3, adjusting for gender, age, complete blood count, liver function, tumor markers, coagulation parameters, scores, and comorbidities). The results showed that ALT was associated with a higher likelihood of recompensation across all 3 models (Model 1: OR = 1.01, 95% CI = 1.00~1.02, *P *= .006; Model 2: OR = 1.01, 95% CI = 1.00~1.02, *P *= .012; Model 3: OR = 1.01, 95% CI = 1.00~1.02, *P *= .007) (Table 3), while qHBsAg was linked to a higher likelihood of recompensation in Model 3 (OR = 1.00, 95% CI = 1.00~1.01; *P*= .037). Two types of variables were used to treat ALT/logqHBsAg. When the ratio was regarded as the continuous variable, in the fully adjusted model, an elevated level of ALT/logqHBsAg was linked to a higher likelihood of recompensation in Model 3 (OR = 1.01, 95% CI = 1.00~1.02; *P *= .027). When the ratio was treated as the categorical variable, ALT/logqHBsAg >23.48 exhibited a higher likelihood of recompensation in different models (Model 1: OR = 3.02, 95% CI = 1.38~6.61, *P *= .006; Model 2: OR = 3.05, 95% CI = 1.28~7.27, *P *= .012; Model 3: OR = 3.21, 95% CI = 1.31~7.90, *P *= .011) (Table 3).

### Subgroup Analyses

**Figure 2.**
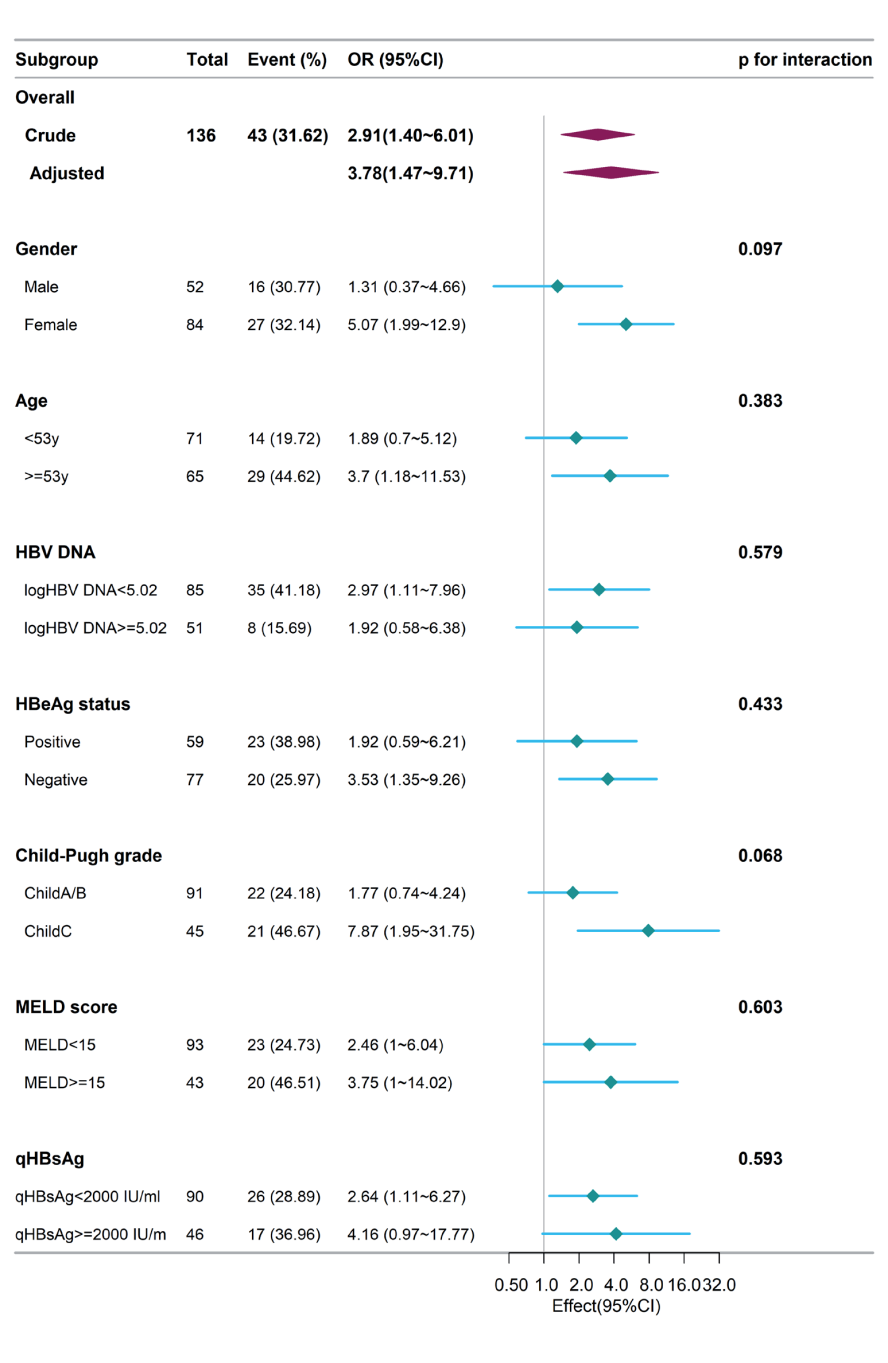
Subgroup analysis: the effect of ALT/logqHBsAg on recompensation.

We further did a subgroup analysis. The outcomes suggested that the influence of ALT/logqHBsAg on the occurrence of hepatic recompensation was uniform among all 7 pre-set subgroups defined by sex, age, HBeAg status, HBV DNA, Child–Pugh grade, MELD score, and qHBsAg (Figure 2). Furthermore, subgroup analyses were performed based on Child–Pugh classification and MELD score to explore whether different subgroups would affect the impact of ALT and qHBsAg on hepatic recompensation (Supplementary Tables 2 and 3). The results showed that the effect of ALT and qHBsAg on hepatic recompensation was consistent across different subgroups.

### Diagnostic Performance of ALT/logqHBsAg

**Figure 3.**
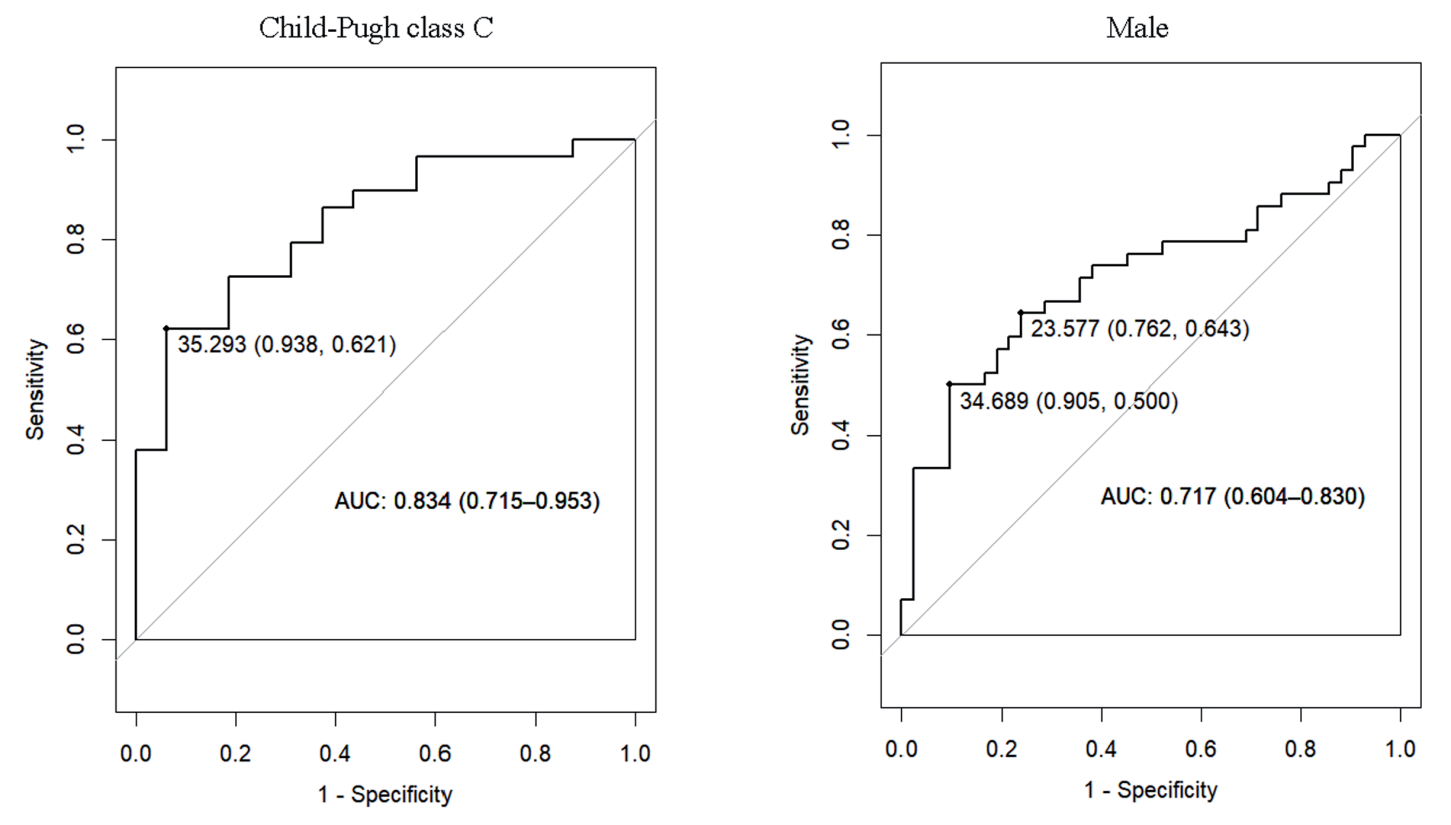
Receiver operating characteristic curves. Diagnostic performance of ALT/logqHBsAg in patients with Child–Pugh class C for recompensation prediction and diagnostic performance of ALT/logqHBsAg in male patients for recompensation prediction.

This ratio exhibited higher predictive performance for cirrhotic recompensation among patients in Child–Pugh class C when contrasted with those in classes A and B, as well as among males when compared to females (Figure 3). Among patients classified as Child–Pugh class C, the ALT/logqHBsAg ratio achieved an area under the receiver operating characteristic curve (AUROC) of 0.834 (95% CI: 0.715-0.953) in predicting cirrhotic recompensation, which outperformed both the MELD score (0.606 [0.423-0.788]) and the FIB-4 index (0.532 [0.364-0.701]). Based on the above results, the AUROC of this ratio in male patients with Child–Pugh class C was further calculated; the results indicated better predictive ability. However, because of the limited sample size within this group (31, 22.8%), the results cannot be generalized for the time being.

## Discussion

Our current study revealed that an elevated level of ALT/logqHBsAg at baseline was linked to an increased probability of hepatic recompensation. In particular, the diagnostic performance of this indicator in predicting cirrhotic recompensation is greater with patients in Child–Pugh class C when contrasted with those in classes A and B. These findings provide new predictive markers for recompensation in patients with decompensated HBV-related cirrhosis and deepen the understanding to a greater extent of the elements that play a role in recompensation.

After treatment according to the cause, liver function reversal may be achieved in patients with cirrhosis at the decompensated stage.^11^^-^^14^ A study in Korea on HBV-related decompensated liver cirrhosis demonstrated that it was more probable for patients with high initial ALT levels to reach the state of cirrhosis recompensation.^8^ Similarly, a study in China on HBV-related decompensated liver cirrhosis suggested that elevated AST levels serve as an independent protective factor for cirrhosis recompensation.^9^ Another study had shown that patients with a baseline MELD score > 15 were more prone to achieve recompensation than those with a MELD score ≤ 15.^15^ Just as previous studies have shown, it was found that when the levels of ALT at baseline were higher, there was an independent association with a higher rate of recompensation after standardized antiviral therapy. This implies that patients with more prominent hepatic necro-inflammatory activity have a greater likelihood of having an effective response to antiviral therapy and attaining liver recompensation.^16^

Chronic HBV infection in the body is a complex and dynamic process. After the virus invades the human body, it involves the interactions between the virus, hepatocytes, and the host’s immune system.^17^ During the process of hepatocyte lysis, ALT is released into the bloodstream. The measured level of ALT in the blood can serve as an important indicator to gauge the degree of liver injury that is mediated by the immune system, while HBsAg and HBV DNA act as proxy markers for transcriptionally active covalently closed circular DNA (cccDNA) and represent a sign for infected hepatocytes.^18^ A research project focusing on liver cirrhosis was carried out in Korea. The researchers employed a competing risk model for univariate analysis. Through this in-depth analysis, they found a correlation: as the level of HBV DNA at the baseline stage increased, the probability of achieving liver recompensation also went up, but in multivariate analysis, this association did not reach statistical significance.^19^ Two other studies from China, including 1 conducted by the team, also yielded similar results.^9^^,^^20^ Until now, no works have addressed the correlation between hepatic recompensation and HBsAg. A recent study revealed that at every time point from the termination of treatment up to week 48, this ratio could independently predict HBsAg seroclearance (HR (1.003-1.028), *P *< .01). As a simple and readily obtainable indicator, the ALT/logqHBsAg can directly reflect the equilibrium state of the host’s immune response to HBsAg. The prognostic significance of ALT/logqHBsAg was assessed for cirrhotic recompensation in the current research. Based on what the authors know, this is the first-ever cohort study which centered on the role of ALT/logqHBsAg in cirrhotic recompensation and found that elevated ALT/logqHBsAg level at baseline was linked to an increased probability of hepatic recompensation. In particular, compared to patients with Child–Pugh A and B, the diagnostic efficacy of this indicator for cirrhotic recompensation is better in patients with Child–Pugh C. This could be attributed to the fact that patients with more severe liver damage tend to exhibit a more robust immune response against the virus, which facilitates better disease prognosis. This process is often accompanied by heightened inflammation and reduced HBV levels. Given that ALT/logqHBsAg effectively integrates information on both liver inflammation and HBV levels, it offers superior predictive value for cirrhotic recompensation in these patients.

Several limitations are acknowledged in this study. Initially, the sample size included in the study was not large enough, and the retrospective nature of the study design could potentially introduce selection bias. However, to mitigate this, 3 multivariate models were employed and subgroup analyses were conducted, all of which consistently produced comparable results. Secondly, patients who achieved recompensation did not undergo liver biopsies. Incorporating liver biopsies could be considered in future prospective studies to verify histological evidence of cirrhosis reversal. Lastly, the HBV genotypes were not examined at the initial phase, but it has been pointed out by prior studies that 85% of HBV genotypes in northern China are genotype C.^21^ Therefore, these findings require further validation in regions with other HBV genotypes.

In conclusion, the higher ratio of ALT to qHBsAg could be used to predict a higher likelihood of recompensation in hepatitis B-related cirrhosis patients based on the latest Baveno VII criteria. Notably, the diagnostic accuracy of this indicator for predicting cirrhotic recompensation is superior in patients classified as Child–Pugh class C. The research findings have provided new, simple, and readily obtainable clinical markers for predicting patients’ recompensation and have enhanced the understanding of the factors influencing recompensation.

## Supplementary Materials

Supplementary Material

## Figures and Tables

**Table 1.** Baseline Characteristics of 136 of the Patients

Variables	Total (n = 136)	ALT/logqHBsAg > 23.48 (n = 77)	ALT/logqHBsAg < 23.48 (n = 59)	*P*
Male sex, n (%)	84 (61.8)	46 (59.7)	38 (64.4)	.579
Age, years	54.6 ± 11.4	56.1 ± 11.2	52.7 ± 11.3	.084
NLR	2.6 ± 2.0	2.6 ± 2.1	2.6 ± 1.8	.836
Platelet, 10^9^/L	88.5 ± 52.1	87.1 ± 60.7	90.3 ± 38.5	.720
ALT, U/L	144.9 ± 285.2	36.3 ± 16.4	286.7 ± 390.9	<.001
AST, U/L	147.6 ± 257.2	51.4 ± 24.3	273.0 ± 353.5	<.001
ALP, U/L	112.0 ± 47.1	100.6 ± 41.6	126.9 ± 50.1	.001
GGT, U/L	74.3 ± 91.2	49.8 ± 50.6	106.4 ± 118.9	<.001
ALB, g/L	30.7 ± 5.6	30.1 ± 5.5	31.5 ± 5.7	.143
TBIL, umol/L	57.8 ± 67.8	41.0 ± 52.7	79.6 ± 78.8	<.001
INR	1.5 ± 0.3	1.4 ± 0.3	1.6 ± 0.3	.005
AFP, ng/mL	363.9 ± 1918.0	257.4 ± 1624.2	502.8 ± 2251.9	.462
HBV DNA, log10 IU/mL	5.3 ± 2.0	5.0 ± 2.1	5.8 ± 1.7	.017
FIB-4	8.5 ± 5.9	7.5 ± 5.0	9.7 ± 6.9	.039
MELD score	14.2 ± 4.7	12.9 ± 3.9	15.9 ± 5.1	<.001
Complications of cirrhosis, n (%)				
Ascites	117 (86.0)	66 (85.7)	51 (86.4)	.904
Esophageal variceal bleeding	14 (10.3)	10 (13)	4 (6.8)	.507
HE	17 (12.5)	8 (10.4)	9 (15.3)	.395
Recompensated cirrhosis	80 (58.8)	37 (48.1)	43 (72.9)	.004
Diabetes	28 (20.6)	17 (22.1)	11 (18.6)	.624

AFP, alpha-fetoprotein; ALB, albumin; ALP, alkaline phosphatase; ALT, alanine aminotransferase; AST, aspartate aminotransferase; FIB-4, Fibrosis-4 Index; GGT, γ-glutamyl transferase; HBV DNA, hepatitis B virus deoxyribonucleic acid; HE, hepatic encephalopathy; INR, international normalized ratio; MELD, model for end-stage liver disease; NLR, neutrophil-to-lymphocyte ratio; TBIL, total bilirubin.

**Table 2.** Logistic Univariate Analysis for Recompensation Prediction

Variables	OR (95% CI)	*P*
Male sex	0.37 (0.17~0.78)	.009
Age, years	0.96 (0.93~0.99)	.007
NLR	0.88(0.74~1.05)	.149
ALT, U/L	1.01 (1.00~1.02)	.004
AST, U/L	1.00 (1.00~1.01)	.030
ALP, U/L	1.00 (0.99~1.01)	.617
GGT, U/L	1.00 (0.99~1.00)	.757
TBIL, umol/L	1.00 (1.00~1.01)	.421
ALB, g/L	1.01 (0.95~1.08)	.697
INR	3.35 (1.05~10.7)	.041
HBV DNA, log_10_IU/mL	1.27 (1.06~1.52)	.010
FIB-4	1.00 (0.94~1.06)	.927
Diabetes	1.10 (0.47~2.58)	.820
qHBsAg, IU/mL	1.00 (1.00~1.01)	.045
ALT/logqHBsAg	1.01 (1.00~1.01)	.032
ALT/logqHBsAg < 23.48	Ref	–
ALT/logqHBsAg > 23.48	2.91 (1.40~6.01)	.004

ALB, albumin; ALP, alkaline phosphatase; ALT, alanine aminotransferase; AST, aspartate aminotransferase; FIB-4, Fibrosis-4 Index; GGT, γ-glutamyl transferase; HBV DNA, hepatitis B virus deoxyribonucleic acid; INR, international normalized ratio; NLR, neutrophil-to-lymphocyte ratio; qHBsAg, quantitative hepatitis B surface antigen; TBIL, total bilirubin.

**Table 3.** Logistic Multivariate Analysis for Recompensation Prediction

	Model 1		Model 2		Model 3	
	OR (95% CI)	*P*	OR (95% CI)	*P*	OR (95% CI)	*P*
ALT	1.01 (1.00~1.02)	.006	1.01 (1.00~1.02)	.012	1.01 (1.00~1.02)	.007
qHBsAg	–	–	–	–	1.00 (1.00~1.01)	.037
ALT/logqHBsAg	–	–	–	–	1.01 (1.00~1.02)	.027
ALT/logqHBsAg>23.48	3.02 (1.38~6.61)	.006	3.05 (1.28~7.27)	.012	3.21 (1.31~7.90)	.011

Model 1, adjusted gender and age-.

Model 2, adjusted gender, age, complete blood count, liver function, and tumor markers.

Model 3, adjusted gender, age, complete blood count, liver function, tumor markers, coagulation parameters, scores, and comorbidities.

ALT, alanine aminotransferase; CI, confidence interval; qHBsAg, quantitative hepatitis B surface antigen.

## Data Availability

The data that support the findings of this study are available on request from the corresponding author.
