# Supplementary material for: The Correlation Between the Ratio of ALT to qHBsAg and the Recompensation of HBV-Related Cirrhosis Patients: A Retrospective Cohort Study Based on the Baveno VII Criteria
Source: Turk J Gastroenterol. 2025 Oct 20;36(11):787–95. doi: 10.5152/tjg.2025.25039 (PMC12616943; doi:10.5152/tjg.2025.25039)
Supplement: Supplementary Material [file supplementary_material.pdf]

Supplementary Table 1. Baseline Characteristics of 136 of the Patients with Decompensated Cirrhosis and Recompensated Cirrhosis

Variables	Total (n = 136)	Recompensated cirrhosis (n = 80)	Decompensated cirrhosis (n = 56)	*p*
Male sex, n (%)	84 (61.8)	42 (52.5)	42 (75)	0.008
Age, y	54.6 ± 11.4	52.4 ± 11.6	57.8 ± 10.2	0.005
Platelet,10^9/L	88.5 ± 52.1	91.1 ± 47.7	84.7 ± 58.0	0.487
ALT, U/L	144.9 ± 285.2	209.5 ± 357.2	52.7 ± 40.2	0.001
AST, U/L	147.6 ± 257.2	197.6 ± 318.3	76.0 ± 89.6	0.006
ALP, U/L	112.0 ± 47.1	113.7 ± 41.2	109.6 ± 54.8	0.620
GGT, U/L	74.3 ± 91.2	76.4 ± 66.3	71.4 ± 118.6	0.758
ALB, g/L	30.7 ± 5.6	30.9 ± 5.7	30.5 ± 5.6	0.699
TBIL, umol/L	57.8±67.8	61.7±67.2	52.1±68.9	0.421
INR	1.5 ± 0.3	1.5 ± 0.4	1.4 ± 0.2	0.036
NLR	2.6 ± 2.0	2.4 ± 1.9	2.9 ± 2.1	0.146
HBV DNA, log10 IU/ml	5.3 ± 2.0	5.7 ± 1.9	4.8 ± 2.0	0.008
MELD score	14.2 ± 4.7	14.7 ± 5.0	13.4 ± 4.1	0.133
FIB4	8.5 ± 5.9	8.4 ± 5.6	8.5 ± 6.5	0.928
HCC, n (%)	17 (12.5)	4 (5)	13 (23.2)	0.002
Diabetes, n (%)	28 (20.6)	17 (21.2)	11 (19.6)	0.82
HBeAg, n (%)				0.002
(-)	59 (44.0)	43 (55.1)	16 (28.6)	
(+)	75 (56.0)	35 (44.9)	40 (71.4)	
qHBsAg, IU/ml	2018.0 ± 3170.7	2518.7 ± 3884.0	1302.6 ± 1457.1	0.027
ALT/log(qHBsAg)	61.1 ± 152.8	87.8 ± 182.0	22.8 ± 84.6	0.014
ALT/log(qHBsAg) > 23.48, n (%)	59 (43.4)	43 (53.8)	16 (28.6)	0.004

Abbreviations: ALT, alanine aminotransferase; AST, aspartate aminotransferase; ALP, alkaline phosphatase; GGT, γ-glutamyl transferase; ALB, albumin; TBIL, total bilirubin; INR, international normalized ratio; NLR, Neutrophil-to-Lymphocyte Ratio; AFP, alpha-fetoprotein; HBV DNA, hepatitis B virus deoxyribonucleic acid; MELD, Model for End-Stage Liver Disease; FIB4, Fibrosis 4 score; HCC, hepatocellular carcinoma; HBeAg, hepatitis B e antigen; qHBsAg, quantitative hepatitis B surface antigen.

Supplementary Table 2. Subgroup analysis of the effect of ALT on recompensation.

Subgroup	Total	Event (%)	OR (95%CI)	*p* for interaction
Child A/B	91	51 (56.0)	1.01 (1~1.01)	0.109
Child C	45	29 (64.4)	1.02 (1~1.04)	
MELD score<15	93	52 (55.9)	1.01 (1~1.02)	0.758
MELD score>=15	43	28 (65.1)	1.01 (1~1.03)	

Abbreviations: MELD score, Model for End-Stage Liver Disease score.

Supplementary Table 3. Subgroup analysis of the effect of qHBsAg on recompensation.

Subgroup	Total	Event (%)	OR (95%CI)	*p* for interaction
Child A/B	91	51 (56.0)	1.01 (1~1.01)	0.280
Child C	45	29 (64.4)	1.01 (1~1.02)	
MELD score<15	93	52 (55.9)	1.01 (1~1.01)	0.944
MELD score>=15	43	28 (65.1)	1.01 (1~1.02)	

Abbreviations: MELD score, Model for end-stage liver disease score.
